# Characterization of Biochars from Different Origins and Their Effects on the Physical-Hydric Properties and Water Use Efficiency in Commercial Substrates

**DOI:** 10.1021/acsomega.6c03840

**Published:** 2026-08-29

**Authors:** Vanessa Susana Rech Bisi, Ruan Fogaça Feijó, Wendel Paulo Silvestre, Marcelo Godinho, Gabriel Fernandes Pauletti

**Affiliations:** † Postgraduate Program in Process Engineering and Technologies (PGEPROTEC), 58802 University of Caxias do Sul, Street Francisco Getúlio Vargas 1130 Petrópolis, Caxias do Sul, RS 95070-560, Brazil; ‡ Laboratory of Plant-Environment Studies (LESPA), University of Caxias do Sul, Street Francisco Getúlio Vargas 1130 Petrópolis, Caxias do Sul, RS 95070-560, Brazil

## Abstract

Biochar has been widely investigated as a soil conditioner due to its potential to improve the relationship between volumetric moisture and water mass, as well as to enhance water use efficiency in agricultural systems. However, there is still limited information regarding its influence on the physical-hydraulic properties of commercial substrates used for seedling production, particularly concerning water retention behavior and practical application in irrigation management. In this study, biochars produced from lemongrass biomass (BCL) and municipal solid waste (MSW) were characterized for physicochemical properties, nutritional composition, and levels of potentially toxic metals. The effect of adding these biochars in different proportions (zero, 5.0%, 7.5%, and 10% m·m^–1^) on the physico-hydric properties and water retention behavior of a commercial substrate used for seedling production was evaluated. The experiment was conducted in pots under controlled conditions, with volumetric moisture and substrate mass measured at successive time points throughout saturation and gravitational drainage. The physical-hydric parameters of the substrate were evaluated, including volumetric moisture, electrical conductivity, water content at saturation, and water content at container capacity, and regression equations were adjusted to estimate available water. The materials exhibited an alkaline character, with pH values of 9.58 for BMSW and 8.83 for BCL, and differences in ash content, ranging from 15.12% m·m^–1^ in BCL to 36.45% m·m^–1^ in BMSW. The nutritional composition indicated the presence of macronutrients, including Ca, Mg, N, K, and P, with higher levels of Ca, N, and P in BMSW and higher levels of Mg and K in BCL. The levels of potentially toxic metals remained below the established limits for agricultural use, indicating environmental safety for application in the substrate. Although overall changes in most physical-hydraulic parameters were limited, biochar incorporation increased container capacity at the highest rates evaluated and enabled the development of a predictive operational model for estimating substrate water retention. This model allows for the estimation of substrate water availability based on volumetric moisture measurements, serving as a practical tool for irrigation management in seedling production systems. Specific differences were observed mainly for volumetric moisture, with values ranging from approximately 0.43 m^3^·m^–3^ in the control substrate to 0.53 m^3^·m^–3^ in the biochar treatments, especially at doses of 7.5% and 10.0% m·m^–1^ of BMSW and at a dose of 10% m·m^–1^ of BCL. The retention curves showed high linearity (R^2^ between 0.962 and 0.998), allowing estimation of the operationally available water in the substrate for water management purposes. It is concluded that biochar can act as a substrate conditioner contributing to the sustainable use of waste and offering a practical approach to irrigation management and increased water use efficiency in container-grown crops, without imposing environmental constraints on the metals evaluated.

## Graphical abstract

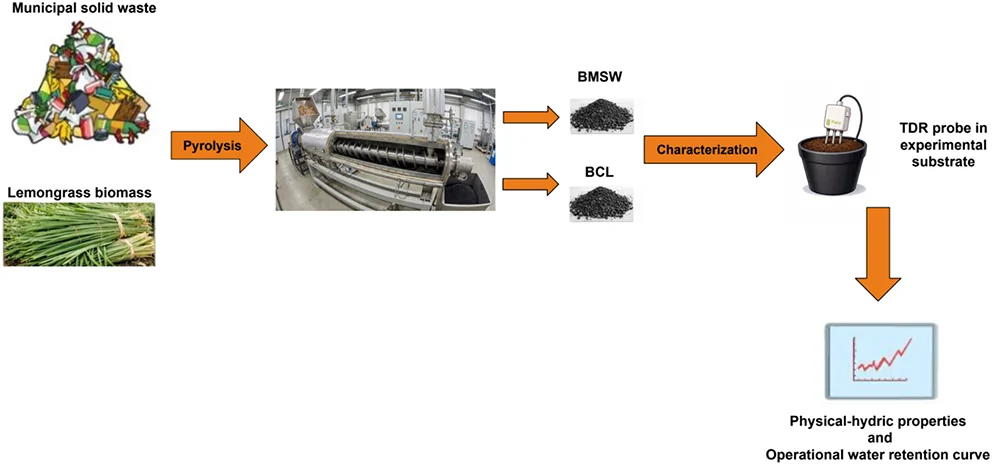

## 1 Introduction

The substrate cultivation method allows control of water availability to plants and the appropriate adjustment of pH and nutrient concentrations in the root zone. Water retention in substrates used in seedling production and cultivation systems is one of the main factors that determine water use efficiency, plant survival, and crop quality.^21,27,9^ The hydric behavior of a substrate is described by water retention curves, which relate volumetric water content to matric potentials or drainage stages, and are a fundamental parameter for characterizing the dynamics of water storage and loss in unsaturated porous media. These curves are essential for understanding the availability of usable water for plants and the medium’s response to variations in environmental water content.^21,9^

With the development of carbon-based materials, there has been a growing demand for alternative applications for these materials. Various studies on biochar have been conducted to improve its physicochemical characteristics, enhance its applications, particularly as a source of energy storage and conversion, and design viable, sustainable applications. Among the possible applications, its use in soil as a conditioner or a soil improver has shown promising results.^10,13,20^

With advances in recent studies, the incorporation of biochar, a carbonaceous material produced by the pyrolysis of biomass under anoxic or low-oxygen conditions, has been widely investigated as a strategy to improve the physical-hydraulic properties of soils and substrates. Biochar has high intrinsic porosity, high specific surface area, macro and microporosity, and chemical properties that influence both the physical structure and the dynamics of water in porous media.^10,13^

The study by^24^ demonstrated that the addition of biochar increases water retention in soils and substrates, especially in coarser-textured soils, due to changes in pore-size distribution and increased microporosity. Complementarily, the meta-analysis by^6^ indicated that biochar application improves hydraulic parameters, including field capacity, plant-available water, and total porosity. However, the intensity of these effects depends on soil type and characteristics, biochar origin, applied dose, and environmental conditions.

However, the effects of biochar on water retention are not uniform. According to,^3^ different feedstocks and application rates may not yield significant increases across the entire soil moisture range, highlighting the complexity of interactions within the soil-plant or substrate-plant system and the need for more detailed investigation.

Furthermore, the biomass source and production conditions—particularly pyrolysis temperature—directly influence biochar’s physicochemical properties, such as porosity, functional groups, and hydrophobicity/hydrophilicity; this results in varying water retention responses following incorporation into substrates.^10,13^ Such variability underscores the importance of studies evaluating different biochar types and proportions, like the one proposed in this work.

In addition to the effects on physical and hydrological properties, the chemical and environmental characterization of biochar is a determining aspect for its agronomic viability, especially regarding nutrient supply and the presence of potentially toxic metals. The elemental composition of biochar is directly related to the raw material used and the pyrolysis conditions. It can result in materials with fertilizing potential as well as products that require restrictions on the applied dose.^13,7^ Studies indicate that biochars tend to have higher levels of ash and mineral elements, including micronutrients and metals, making it essential to compare them with limits established by environmental legislation to ensure their safe application in soil and agricultural substrates.^9,29^

The selection of the biomasses used in this study was based on their environmental relevance, availability, and potential for valorization within the circular economy framework. Biochar produced from lemongrass biomass offers an alternative for utilizing agro-industrial residues generated after essential oil extraction, thereby adding value to a material that typically has limited disposal options. This biomass originates from an experimental area dedicated to research on aromatic plants.

Meanwhile, biochar derived from municipal solid waste is part of a resource recovery strategy involving the thermochemical conversion of such waste, aiming to reduce the volume sent to landfills. Thus, these biochars represent two distinct waste categories—agro-industrial and municipal—enabling an assessment of how biomass origin influences the physicochemical properties of the biochar and its performance in commercial growing media. Furthermore, the use of these biomasses supports circular economy strategies by promoting waste valorization through the production of higher-value materials for horticulture and sustainable water management in container-based cultivation systems.

It is also worth noting that the operational approach to water retention proposed in this study offers a practical advantage over traditional curves based on matric potential, as it allows for the rapid estimation of substrate water availability through direct volumetric moisture measurements. Unlike conventional methods, which require specialized laboratory equipment, pressure plates, and lengthy analysis periods, the methodology employed here enables substrate water monitoring using TDR sensors, which are widely applicable in commercial production settings. In this context, the proposed method offers a simpler, faster, and operationally feasible alternative for irrigation management in container-based cultivation systems.

Despite the growing number of studies on agricultural soils related to biochar, there is relatively little research specifically dedicated to the influence of biochar addition in commercial substrates used in protected cultivation or seedling production, especially through detailed analysis of saturation and drainage curves under different biochar doses. Most studies were conducted on agricultural soils, whereas commercial substrates have completely different characteristics (high porosity, high water retention, and organic composition). This represents a significant gap, since substrates and soils exhibit distinct hydraulic behaviors due to differences in their texture, porosity, and organic and mineral composition. Therefore, understanding how biochars from different source materials and in different proportions alter the operational water retention curve in these systems is essential to maximize the water performance of substrates in agricultural and horticultural applications, aiming at the rational and sustainable use of water resources and soil/substrate.

Given this gap in understanding biochar behavior within commercial substrates and the hypothesis that different feedstocks can differentially influence the physical-hydraulic properties and water retention of these systems, this study aimed to evaluate the effect of incorporating biochar—produced from lemongrass residual biomass and municipal solid waste (MSW) at various rates (zero, 5.0, 7.5, and 10 wt %)—on the physical-hydraulic properties and water retention curves of a commercial substrate, seeking to understand the interactions between biochar origin and the substrate’s hydraulic behavior.

## 2 Materials and Methods

### 2.1 Materials

#### 2.1.1 Biochars

The lemongrass biomass biochar was obtained from the residue generated after essential oil extraction in experiments conducted at the institution’s aromatic plant research facility. The MSW biochar originated from a project aimed at evaluating pyrolysis as an alternative for the valorization and recovery of resources contained in such waste.

Biochars were obtained by slow pyrolysis of lemongrass and MSW biomass at 350 and 450 °C, respectively. The temperatures were obtained from previous studies conducted by Bisi,^37^ considering the intrinsic characteristics of the biomasses. The biochars produced at these temperatures allow for an assessment of how materials with varying degrees of carbonization might influence the physical-hydraulic properties of the substrates; this is because the distinct pyrolysis temperatures reflect typical conversion conditions for each biomass and were selected to optimize biochar stability.

The biomass was pyrolyzed in an Auger-type reactor at a heating rate of 10 °C·min^–1^ and a residence time of approximately 90 min. After cooling, the material was ground in a hammer mill and sieved through a 2.0 mm sieve.

The characterization of the biochar was conducted to evaluate its physicochemical properties and potential for use in substrate composition. Immediate analysis, pH evaluation, electrical conductivity, and density were performed, as well as the evaluation of surface area and porosity by the Brunauer, Emmet, and Teller (BET) method, and scanning electron microscopy (SEM). Macro- and micronutrient content, as well as the presence of heavy metals, were assessed. For the results relating to proximate analysis, pH evaluation, electrical conductivity, and biochar density, the averages of triplicate assays are presented.

Immediate analysis was performed to determine the moisture, ash, volatile matter, and, by difference, fixed carbon content of the material. Moisture content was determined according to ASTM D3173–11. The difference between the initial and final masses of the samples obtained was the moisture content. Ash analysis followed the procedure described in ASTM D3174–12, and volatile matter was determined according to ASTM D3175–13. Fixed carbon content was calculated using mass balance, according to ASTM D3172–13. Electrical conductivity (EC) and pH of the biochar samples were determined according to ASTM D4980–89.

To determine the density of the biochars, the sample volume was measured by sieving a specific quantity of biochar through a 200-mesh sieve in a calibrated 10 mL soil sampler. Subsequently, the measured 10 mL was weighed on an analytical balance. The density was calculated in triplicate by dividing the sample mass by its volume.

For the analysis of macronutrients Ca, Mg, N, K and P and micronutrients Cu and Zn present in the biochars, the methodology of^17^ was adopted and the analysis of the total contents of metals As, Cd, Pb, Cu, Cr, Hg, Mo, Ni, and Zn was carried out using methods 3050*B*/1996 of the Environmental Protection Agency (EPA) and Method 999.11/2006 of the Association of Official Analytical Chemists (AOAC).

For the BET test, the sample was initially degassed in an inert atmosphere (nitrogen) at 250 °C for 24 h to remove surface contaminants. Subsequently, the sample was analyzed for pore size and surface area using Quantachrome Instruments equipment (model 1200e).

The SEM (Scanning Electron Microscopy) assay was performed to evaluate the morphology of the materials. Initially, the samples were placed in a desiccator for 24 h to remove residual moisture. Then, approximately 1.0 g of biochar, previously sieved through a 200-mesh sieve, was placed on an aluminum support and fixed with conductive carbon tape. Images were obtained using a scanning electron microscope operating at 10 kV, with magnifications of 250× and 1000×.

#### 2.1.2 Commercial Substrate Characterization

The experimental substrate used as the growing medium is widely employed in the production of vegetable seedlings and is composed predominantly of organic materials characterized by high porosity and high water-holding capacity. This base substrate was selected due to its widespread use in protected cultivation systems, allowing for the evaluation of biochar’s effects under conditions that closely resemble actual commercial practices. The commercial substrate, Carolina Soil, used in experiments, is commonly used in commercial nurseries for seedling production. It is characterized as a lightweight, contaminant-free substrate composed of sphagnum peat moss, expanded vermiculite, roasted rice husk, and hydrofiber. The pH of the pure material is 5.5, and its electrical conductivity is 0.7 mS·cm^–1^, according to the supplier.

### 2.2 Methods

#### 2.2.1 Substrate Preparation and Saturation

Initially, the 240 cm^3^ plastic pots containing the substrate were weighed. Then, the pots were filled with commercial substrate and the respective amounts of MSW biochar and lemongrass biochar, at zero, 5.0, 7.5, and 10.0 wt % of biochar, considering previous studies conducted by Bisi.^37^ Furthermore, these proportions are considered technically feasible for commercial-scale application, allowing for the analysis of the incremental effect of biochar on the physical and hydraulic properties of the substrate.

The mixtures were prepared individually for each treatment, ensuring complete homogenization of the biochar with the commercial substrate before the start of the experimental evaluations. The experimental design consisted of seven treatments, with three replicates per treatment, for a total of 21 pots placed in a plastic tray for saturation. The experiment was conducted in a completely randomized design in a factorial scheme, considering two types of biochar and four incorporation proportions. After placing the substrate in the pots without compacting the material to simulate real-world use, the pots were weighed and saturated. Water was slowly added to the plastic tray until it reached the surface of the pot, filling the substrate’s pores. After the substrate’s complete saturation, the water was removed from the plastic tray, and the containers were immediately weighed and placed back in the tray for gravity drainage for 24 h. This condition represents the container capacity, i.e., the water remaining in the substrate after gravity drainage.

At complete saturation, volumetric moisture content (VMC) and electrical conductivity (EC) were measured using a Meter Em50 TDR probe, and the pots were weighed on an analytical balance. These measurements were taken daily until the pots reached approximately 50% of their saturated weight, as measured by the VMC TDR probe, to determine the substrate’s container capacity. Subsequently, the pots containing the substrate were dried in a forced-air oven at 105 ± 5 °C for 24 h to obtain the final dry mass.

After obtaining the substrate masses under different water conditions, calculations were performed for the water content at saturation and the water content at container capacity, defined in this study as the substrate condition after gravity drainage. This approach is widely used for the physical-hydrological characterization of horticultural substrates, as described by ref 5 and later discussed in a study on the water–air relationship in substrates for propagation.^32^

Based on the values obtained during the various drainage stages, the substrate’s operational water retention curve was constructed by relating the mass of water retained in the substrate to the corresponding volumetric water content, adapting the methodology described in ref 19. This approach allows for a direct correlation between substrate mass variations and the volumetric water content measured by TDR sensors throughout the saturation and drainage stages. It should be noted that the curve obtained in this study represents an operational water retention approach—based on the relationship between water mass and volumetric water content—rather than the classic retention curve plotted against matric potential; this eliminates the need to determine curves using laboratory pressure equipment.

The operational water retention curve was plotted from the saturation condition to the last experimental measurement, allowing evaluation of the substrate’s water behavior throughout the water-loss process and determination of the material’s container capacity. The equation of the line was generated in LibreOffice Calc to obtain the volume of water in the substrate relative to a given measured volumetric moisture content, assuming a specific mass of water of 1.0 g·mL^–1^.

### 2.3 Statistical Analysis

The data were subjected to analysis of variance (ANOVA) in a completely randomized design, considering a 2 × 4 factorial scheme (two types of biochar × four incorporation doses), verifying the significance of the main effects (type of biochar and dose) and the interaction between the factors using the *F*-test (*p* ≤ 0.05). The means were compared using Tukey’s test at a 5% probability level. Statistical analyses were performed using the AgroEstat program (Brazil).

## 3 Results and Discussion

### 3.1 Characterization of Biochar

#### 3.1.1 Immediate Analysis, pH, Electrical Conductivity, and Density

The results obtained for each parameter related to the immediate analysis and the analyses of pH, conductivity, and density of MSW and lemongrass biochar are presented in Table 1.

**Table 1:** Results of pH, Conductivity, Density, and Proximate Analysis of Biochars from Municipal Solid Waste (MSW) and Lemongrass Biomass^a^

Parameter	Unit	MSW biochar	Lemongrass biochar
pH	-	9.58 ± 0.02	8.83 ± 0.07
electrical conductivity	dS·m^–1^	8.37 ± 0.13	4.18 ± 0.05
Moisture	% m·m^–1^	1.41 ± 0.01	4.01 ± 0.06
Ash	% m·m^–1^	36.45 ± 0.06	15.12 ± 0.01
Volatile material	% m·m^–1^	28.45 ± 0.02	40.22 ± 0.01
Fixed carbon	% m·m^–1^	33.73 ± 0.18	40.65 ± 0.18
Density	kg·m^–3^	440.00 ± 10.00	192.00 ± 10.00

a Source: prepared by the authors, 2026.

The results of the physicochemical characterization of biochars produced from municipal solid waste and lemongrass reveal important differences associated with the nature of the original biomass, which directly influence the behavior of these materials when applied as substrates and/or soil conditioners.

The pH of the biochars was in the alkaline range, being 9.58 for MSW biochar and 8.83 for lemongrass biochar. These values are within the range typically reported for biochars produced at intermediate temperatures, especially those derived from biomasses with higher mineral content, whose pH values generally range between 7–10.^14,31^ The more pronounced alkalinity of MSW biochar can be attributed to its higher ash content, which indicates a greater presence of hydroxides, oxides, carbonates, and alkaline salts formed during pyrolysis, as described in the literature by refs 22,29 .

Electrical conductivity (EC) showed values of 8.37 dS·m^–1^ for MSW biochar and 4.18 dS·m^–1^ for lemongrass biochar, indicating a higher concentration of soluble salts in MSW biochar. Studies report that the EC of biochars can vary widely, depending on the source material and its degree of mineralization.^22,7^ Thus, the values observed in this study are within the range reported in the literature. On the other hand, the higher EC of MSW biochar suggests caution in its application to substrates, given its greater salinity tolerance, as described by ref 36.

Regarding moisture content, the observed values were 1.41% m·m^–1^ for MSW biochar and 4.01% m·m^–1^ for lemongrass biochar, which is consistent with the typical behavior of freshly produced biochars, characterized by low moisture content due to the removal of structural and bound water during pyrolysis.^13^ The higher moisture content in lemongrass biochar may be associated with its lower density and higher internal porosity, which favor water retention in micropores and residual cellulose and lignin hydrolysis.^12^

Regarding the immediate analysis, MSW biochar presented 28.45% m·m^–1^ of volatile material, 33.73% m·m^–1^ of fixed carbon, and a high ash content. In comparison, lemongrass biochar presented 40.22% m·m^–1^ of volatile material and 40.65% mm^–1^ of fixed carbon. These results indicate that lemongrass biochar tends to have a higher fraction of fixed carbon, indicating a greater presence of relatively stable carbonaceous material, a characteristic common to biochars derived from lignocellulosic biomass, even when produced at lower pyrolysis temperatures, such as 350 °C.^7^ The higher fraction of fixed carbon in lemongrass biochar suggests greater structural stability and a potentially greater contribution to improving the substrate’s physical properties.^18^

The ash content showed a significant difference between the materials, being 36.45% m·m^–1^ in MSW biochar and 15.12% m·m^–1^ in lemongrass biochar. Biochars derived from urban waste and sludge are frequently described as materials with high ash content, which can exceed 30% m·m^–1^, while biochars of plant origin generally present values lower than 20% m·m^–1^. It is worth noting that lower ash contents tend to indicate greater carbon fixation in the biochar matrix.^7^ In this context, the results align with the literature and partially justify the higher pH and electrical conductivity observed in MSW biochar.

The apparent density of the biochars also showed marked differences, being 440 kg·m^–3^ for MSW biochar and 192 kg·m^–3^ for lemongrass biochar. Similar values are reported in the literature, which describe mineralized biochars as denser, while plant biomass-derived biochars have lower densities, associated with greater porosity.^22^ Thus, the lower density of lemongrass biochar suggests greater potential to improve aeration and water retention when incorporated into agricultural substrates.^16^

#### 3.1.2 Surface Area and Porosity of Biochars

According to BET analysis, the surface area of MSW biochar and lemongrass biochar corresponds to 7.56 m^2^·g^–1^ and 4.92 m^2^·g^–1^, respectively. It is important to emphasize that the larger the material’s contact surface, the greater the area for chemical interactions. The average pore diameter and volume obtained by BET analysis for the biochar samples are presented in Table 2.

**Table 2:** Average Diameter and Total Pore Volume of *Biochars* from Municipal Solid Waste and Lemongrass Biomass^a^

Material	Average pore diameter (nm)	Total pore volume (cm^3^·g^–1^)
MSW biochar	12.90	1.31 × 10^–2^
Lemongrass biochar	8.76	4.45 × 10^–3^

a Lemongrass biochar was pyrolyzed at 350 °C, while MSW biochar was pyrolyzed at 450 °C. Source: Authors, 2026.

Regarding pore diameter, it is noted that, according to,^30^ the observed pores fall mostly within the mesopore range, which plays a relevant role in the capillary retention of water available to plants. The effect of pyrolysis temperature on the average pore diameter and volume can be observed in the increase in average pore diameter and total pore volume with increasing temperature, as lemongrass biochar was pyrolyzed at a lower temperature than MSW.

MSW biochar had the largest volume and average pore diameter among the biochars, which may be associated with the different biomasses and inorganic fractions present in the materials.^8^ highlight that increasing the temperature tends to increase the amount of micropores in the material.^23^ emphasizes that the microporosity of biochars can favor the adsorption and transport of molecules, as well as the biochar’s interactions with the medium.

Different authors highlight that biochar porosity is associated with sorption capacity, improving water availability, and serving. These authors also report that the pores play an important role in water retention and adsorption processes in the soil.^33,25,34,23^

Scanning electron microscopy (SEM) analysis was performed on both biochars, and the results are presented in Figure 1, which shows images obtained at 250× magnification. The materials exhibit heterogeneous morphology, with particles of varying granulometry.

**Figure 1:**
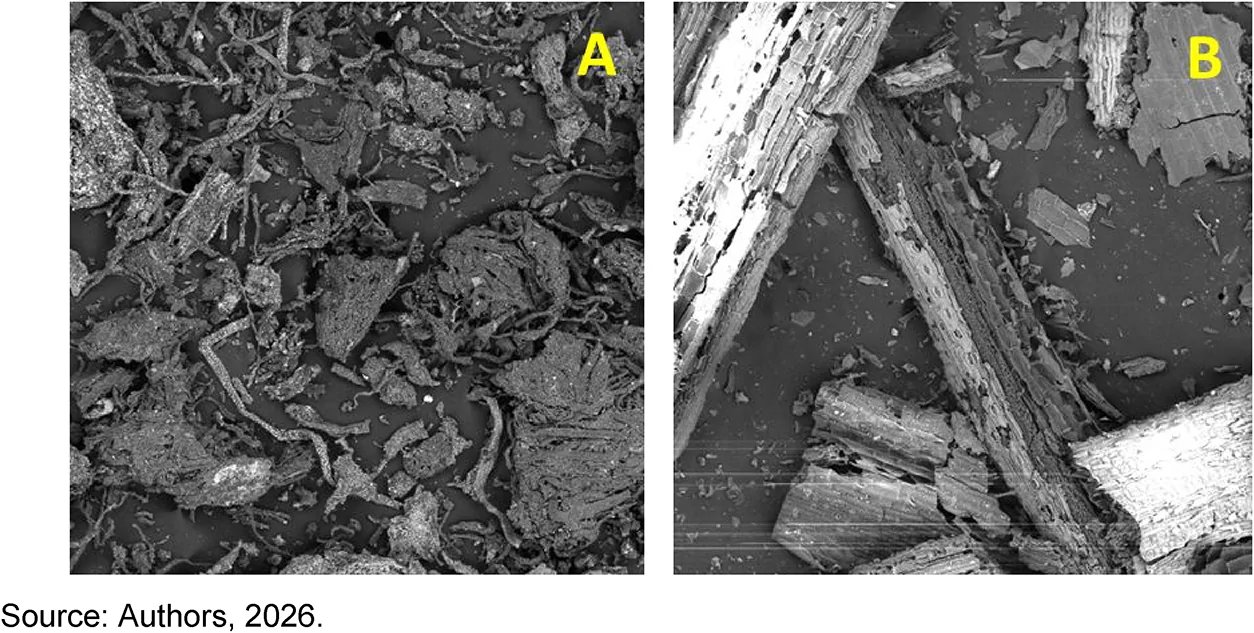
SEM images of MSW biochar (A) and lemongrass biochar (B) at 250× magnification. Source: Authors, 2026. The surface morphology and porosity of the biochar particles from MSW (A) and lemongrass (B) are shown in Figure 2, obtained at 1000× magnification.

SEM analysis revealed that the biochars exhibit heterogeneous morphology, with particles of varying granulometry and irregular surface structures. This structural heterogeneity is a common characteristic of biochars produced from different raw materials and pyrolysis conditions, since the thermal decomposition of biomass partially preserves cellular structures of the original material, resulting in particles with varying sizes and shapes. Furthermore, the particle size distribution and morphology can directly influence relevant physical properties, such as porosity and specific surface area Figure 2.^12,23^

**Figure 2:**
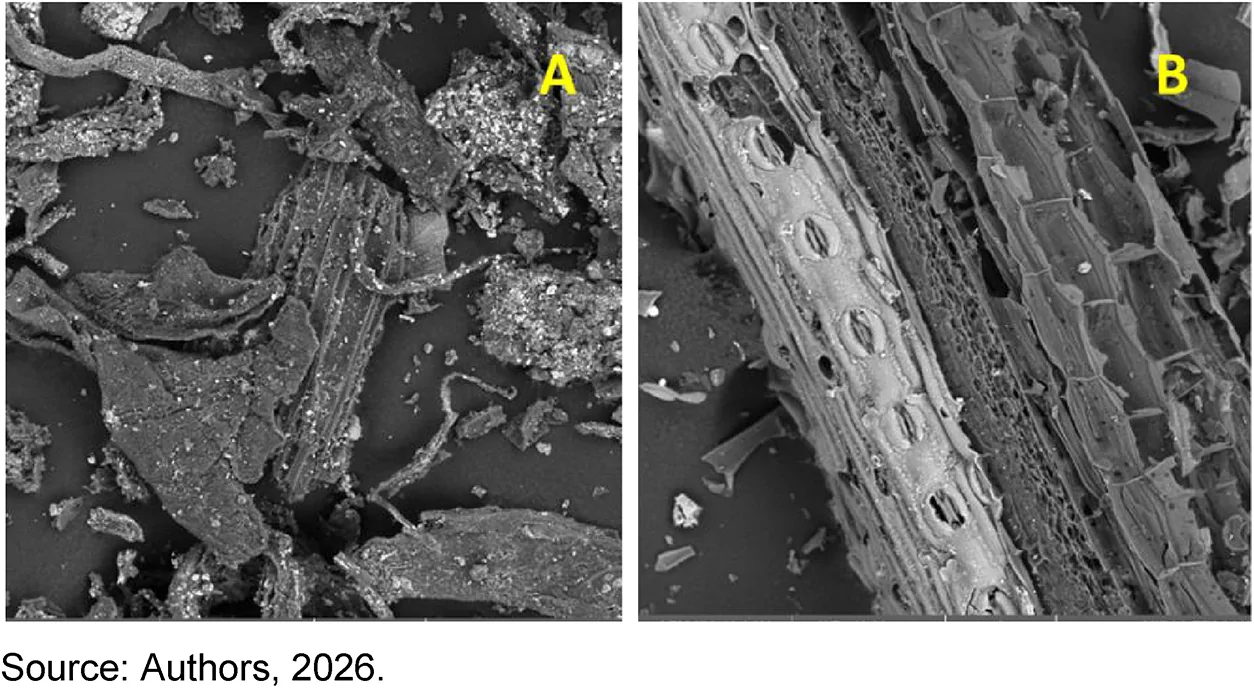
SEM images of MSW biochar (A) and lemongrass biochar (B) at 1000× magnification. Source: Authors, 2026.

In the micrographs at higher magnification (1000×), small pores distributed on the particle surfaces are observed. The porous structure is considered one of the main characteristics of biochar, responsible for its high surface area and the capacity to adsorb water and nutrients. According to,^8^ a smaller quantity of visible pores may be associated with damage to the micropore structure or the incomplete formation of these structures during the pyrolysis process.

The literature indicates that these pores span different scales, from micropores to macropores, and their formation is directly related to both the biomass’s anatomical structure and the pyrolysis process conditions.^30^ This porous structure can enhance water retention and nutrient adsorption when biochar is incorporated into soil or agricultural substrates, thereby improving the physical and chemical properties of the growing medium.^15,26^ Furthermore, studies indicate that the high porosity and large surface area of biochar are directly associated with increased water retention capacity and improved hydrological properties of the soil or substrate.^4,15,23^

#### 3.1.3 Nutrient and Heavy Metal Content in Biochars

The results obtained for the levels of macronutrients Ca, Mg, N, K, and P and micronutrients Cu and Zn present in MSW and lemongrass biochar are presented in Table 3, compared to the levels of these elements present in organic fertilizers such as cured bovine manure, chicken manure, sewage sludge, and compost, reported by ref 28.

**Table 3:** Nutrient Content in MSW and Lemongrass Biochars Compared with that of Some Organic Fertilizers Reported in the Literature

Material	N (g·kg^–1^)	Ca (g·kg^–1^)	Mg (g·kg^–1^)	K (g·kg^–1^)	P (g·kg^–1^)	Cu (mg·kg^–1^)	Zn (mg·kg^–1^)
MSW biochar	18.8	59.4	3.4	14.5	8.3	38.7	587.6
Lemongrass biochar	13.3	9.2	5.7	38.0	4.0	41.9	85.1
Cured cow manure^a^	15.0	20.0	6.0	21.0	12.0	25.0	217.0
Chicken manure^a^	14.0	23.0	5.0	7.0	8.0	14.0	138.0
Sewage sludge^a^	16.0	16.0	6.0	2.0	8.0	435.0	900.0
Waste compost^a^	6.0	11.0	1.0	3.0	2.0	107.0	255.0

a Results extracted from ref. 28. Source: Authors, 2026.

As shown in Table 3, the evaluated biochars, compared with organic fertilizers traditionally used in agriculture, exhibit a nutritional composition that indicates potential for use as soil conditioners. According to, ref 29 materials that have the capacity to add nutritional elements to the soil have agronomic viability for use, either as fertilizer or as a conditioner, contributing to the replenishment and balance of nutrients in the soil-plant system.

MSW biochar showed higher levels of calcium, nitrogen, and phosphorus than lemongrass biochar, highlighting its greater potential as a complementary source of these macronutrients. In contrast, lemongrass biochar showed higher levels of magnesium and potassium, indicating that the plant raw material favors the concentration of these elements in the pyrogenic material. In a study conducted by ref 8 with elephant grass biochars, the authors observed increases in the levels of phosphorus, potassium, calcium, and magnesium in the soil after the application of the biochar, also noting that lignocellulosic biochars tend to have lower nitrogen levels due to the high pyrolysis temperatures, associated with the loss of volatile compounds.

The Cu levels observed in the biochars were lower than those reported for sewage sludge and composted waste.^28^ For zinc, MSW biochar showed the highest level among the biochars analyzed, although this value is lower than that observed for sewage sludge, as reported by ref 28. It should be noted that lemongrass biochar showed the lowest Zn levels among the materials evaluated.

The results for the determination of heavy metals in MSW and lemongrass biochar are presented in Table 4 and compared with the maximum limits set by the Conama Resolution 375/2006.

**Table 4:** Heavy Metal Content of MSW and Lemongrass Biochars, Compared to the Maximum Concentration Allowed by Conama Resolution 375/2006^a^

	MSW biochar	Lemongrass biochar	Maximum permitted concentration
Element	mg·kg^–1^	mg·kg^–1^	mg·kg^–1^
As	<0.52	<0.52	41
Cd	0.74	0.23	39
Pb	20.20	3.05	300
Cu	38.67	41.89	1,500
Cr	36.12	3.92	1,000
Hg	<0.15	<0.15	17
Mo	10.53	1.40	50
Ni	33.62	2.35	420
Zn	587.60	85.10	2,800

a Source: Authors, 2026.

Based on the results presented in Table 4, no critical levels of metals were observed that would make the application of biochars to the soil unfeasible, considering the maximum concentrations allowed by current legislation. However, the maximum application dose of these materials must be established so as not to exceed the legal limits for each element. Thus, it is necessary to consider the element present at the highest concentration in each biochar as a limiting factor for the applied dose, to avoid potential risks of soil contamination from excessive use of the material.

### 3.2 Physical-Hydric Properties and Saturation Curve of the Substrate

#### 3.2.1 Physical-Hydric Parameters of a Commercial Substrate with the Addition of Different Biochars

The physical-hydric properties of substrates containing biochar are directly related to the structural and physicochemical characteristics of the incorporated material. In the present study, the biochars presented important differences in density, specific surface area, and pore volume, characteristics that can influence water retention and redistribution in the substrate-biochar system. In general, materials with higher intraparticle porosity and high surface area tend to favor capillary water retention and the formation of microreservoirs in the porous medium. Thus, the interpretation of the physical-hydric results presented below should consider these structural characteristics previously observed in the characterization of the biochars.

Table 5 presents the results for volumetric moisture (m^3^·m^–3^), electrical conductivity (mS·cm^–1^), water content at saturation, and water content at container capacity for a commercial substrate treated with different doses of lemongrass biochar and municipal solid waste (MSW) biochar.

**Table 5:** Physical-Hydric Parameters of a Commercial Substrate with the Addition of Different Concentrations of Lemongrass Biochar and Municipal Solid Waste (MSW)^a^

Treatment	Volumetric moisture (m^3^·m^–3^)		Electrical conductivity (mS·cm^–1^)		Water content at saturation (g_H_2_O_·g_substrate_^–1^)		Water content in a container capacity (g_H_2_O_·g_substrate_^–1^)	
	BCL	BMSW	BCL	BMSW	BCL	BMSW	BCL	BMSW
Control	0.43 ± 0.04Ab	0.43 ± 0.04Ab	0.31 ± 0.03Ab	0.31 ± 0.03Ab	3.29 ± 0.31Aa	3.29 ± 0.31Aa	3.04 ± 0.26Ab	3.04 ± 0.26Ab
5.0 wt %	0.43 ± 0.07Bab	0.49 ± 0.04Aab	0.37 ± 0.05Aa	0.38 ± 0.06Aa	3.71 ± 0.62Aa	3.78 ± 0.61Aa	3.34 ± 0.43 Aab	3.12 ± 0.14Aab
7.5 wt %	0.45 ± 0.01Bab	0.53 ± 0.01Aa	0.35 ± 0.02Aab	0.32 ± 0.01Aab	3.67 ± 0.17Aa	3.90 ± 0.24Aa	3.43 ± 0.12 Aab	3.32 ± 0.18Aab
10.0 wt %	0.49 ± 0.03Ba	0.52 ± 0.01Aa	0.36 ± 0.04Aab	0.37 ± 0.01Aab	3.87 ± 0.06 Aa	3.71 ± 0.14Aa	3.57 ± 0.05Aa	3.43 ± 0.04Aa
CV	7.60		10.72		9.96		6.69	

a Lowercase letters in the same column indicate a significant difference between treatments according to Tukey’s test (*p* ≤ 0.05). CV: coefficient of variation. BCL: lemongrass biochar; BMSW: municipal solid waste biochar. Source: Authors, 2026.

Regarding volumetric moisture, values increased with biochar addition, especially in treatments with MSW biochar. The highest value was recorded in the treatment with 7.5 wt % of MSW biochar (0.53 m^3^·m^–3^), followed by the treatment with 10% m·m^–1^ of the same biochar (0.52 m^3^·m^–3^). These treatments showed higher values than the control substrate (0.43 m^3^·m^–3^), although the statistical difference is observed mainly between the higher doses and the control.

For lemongrass biochar, only the 10 wt % dose showed a significant increase in volumetric moisture compared to the control, while the 5 and 7.5 wt % doses did not differ statistically. Despite these punctual increases, no linear or proportional behavior of volumetric moisture was observed as a function of the increasing biochar dose in the MSW treatment.

EC values ranged from 0.31 mS·cm^–1^ in the control substrate to 0.38 mS·cm^–1^ in the treatment with 5% mm^–1^ of MSW biochar and 0.37 mS·cm^–1^ in the treatment with 5% mm^–1^ of lemongrass biochar. Although these values are statistically higher than the control, the observed differences do not indicate a significant alteration in the EC of the substrate under the evaluated experimental conditions. These variations may be associated with the release of soluble ions present in the biochar itself, depending on the biomass source and pyrolysis conditions, as reported by refs 7,29 .

The water content parameters at saturation did not show statistically significant differences between treatments, indicating that, at the evaluated biochar doses (5% to 10% m·m^–1^), there was no consistent impact on the substrate’s water retention capacity at saturation. Similar results have been reported in studies that evaluated the incorporation of biochar in organic substrates and soils with high initial porosity, in which the addition of the material does not promote significant changes in water retention under saturation conditions, since the system already has a high fraction of macropores.^11,2^ In these cases, the effects of biochar tend to manifest mainly in the redistribution of pores responsible for capillary water retention, rather than in an increase in total water retained at saturation.

However, regarding the water content in the container capacity, a distinct behavior was observed. The control substrate presented the lowest values (3.04 g_H_2_O_·g_substrate_^–1^), differing statistically from the treatments with 10% m·m^–1^ of biochar, both lemongrass and MSW, which presented the highest values (3.57 g_H_2_O_·g_substrate_^–1^ and 3.43 g_H_2_O_·g_substrate_^–1^, respectively). The doses at 5% and 7.5% m·m^–1^ showed intermediate values, without statistically differing from the control or the highest dose, suggesting a tendency for increased container capacity with increasing biochar dose.

The increased water retention after free drainage in treatments with higher doses of biochar has been frequently associated with the presence of micro- and mesopores in the carbonized material’s structure, which favors capillary water retention in the substrate-biochar system.^12,22^ These internal pores in particles can act as additional water reservoirs, helping maintain moisture available to plants.

The interpretation of these results can be linked to the structural characteristics of the biochars obtained during the physicochemical characterization. As shown in Table 2, the MSW biochar presented a larger average diameter and greater total pore volume compared to the lemongrass biochar, which may favor water storage in intraparticle pores. Pores in the mesopore range (2–50 nm), as observed in this study, are particularly relevant for capillary water retention in porous materials, contributing to water storage after gravitational drainage. Thus, the presence of these pores in the biochar may act as microreservoirs of water in the substrate-biochar system, explaining the higher volumetric moisture values observed mainly in treatments containing MSW biochar.

These results suggest that although the addition of biochar did not significantly alter the water content at saturation, the application at a 10% m·m^–1^ dose increased the substrate’s capacity to retain water after free drainage. This behavior may be associated with modifications in the pore-size distribution in the substrate-biochar system, resulting from the incorporation of biochar particles with high intraparticle porosity and specific surface area, and with an increase in the fraction of pores responsible for capillary water retention, an effect more evident at higher doses of the material.

Another factor that may have contributed to this behavior is the difference in apparent density between the biochars evaluated. Lemongrass biochar had a significantly lower density (192 kg·m^–3^) than MSW biochar (440 kg·m^–3^), a characteristic often associated with a higher fraction of internal pores and less particle compaction in the substrate. Less dense biochars tend to increase the total porosity of the medium and favor water retention in porous systems, which may contribute to the observed increases in volumetric moisture in the treatments with higher doses.

It is worth noting that the main mechanism by which biochar can modify the water properties of a medium to which it is added is through alteration of the pore structure, both in intraparticle pores (within the biochar) and in the pores between particles of the substrate-biochar system, increasing capillary retention capacity and the surface area available for water adsorption.^12,2^ However, this improvement often depends on the combination of biochar and soil or substrate type, since in finer substrates or those already rich in organic matter and porosity, the effects may be less pronounced, which may explain the absence of significant responses in the classic physical-hydric parameters evaluated between treatments.^1,26,22,2^

In addition, several studies have shown that the impact of biochar on soil or substrate water properties depends strongly on the interaction between its physical characteristics and the medium’s structure.^7,10^ Biochars produced from different source materials and at different pyrolysis temperatures exhibit significant variations in porosity, surface area, and hydrophobicity, factors that directly influence the system’s water behavior.^7,10^

In contrast,^26^ observed, in a meta-analysis, that coarse-textured (sandy) soils tend to increase water storage capacity by more than 71% in greenhouse and pot studies when biochar is added. According to the study, this occurs due to changes in porosity and increased water storage capacity, which reduce apparent density and increase the pore space available for water retention.

[1] found that applying biochar can increase water-holding capacity, especially in sandy soils. However, these same authors emphasize that this impact cannot always be observed, as it depends on the biochar characteristics resulting from the pyrolysis conditions and the biomass of origin.

Thus, the response of physical-hydraulic properties to biochar application can be highly variable, depending on the raw material, pyrolysis temperature, and physical-chemical characteristics of the biochar.^1^ More porous biochars with a larger surface area tend to have a greater impact on water retention, especially in sandy soils, than biochars with lower porosity or derived from denser materials, especially when applied to finer textured soils.^26^

Thus, the variations observed in this study for treatments with different biochars and different application rates may reflect not only the effect of the addition, but also the intrinsic characteristics of these materials, since changes in physical-hydric properties after biochar application can vary widely depending on the type of soil or substrate, the biochar production conditions, and the applied dose.

#### 3.2.2 Evaluation of the Operational Water Retention Curve of a Commercial Substrate with Added Biochar

The relationship between volumetric moisture and the mass of water retained in the substrate was developed from measurement data, assuming a specific mass of water of 1.0 g·mL^–1^. The generated linear regression curves are presented in Figure 3. Given that the 2 × 4 factorial analysis did not show statistically significant differences in biochar or concentration, the generated curves are comparable between the pure substrate and the two biochars.

**Figure 3:**
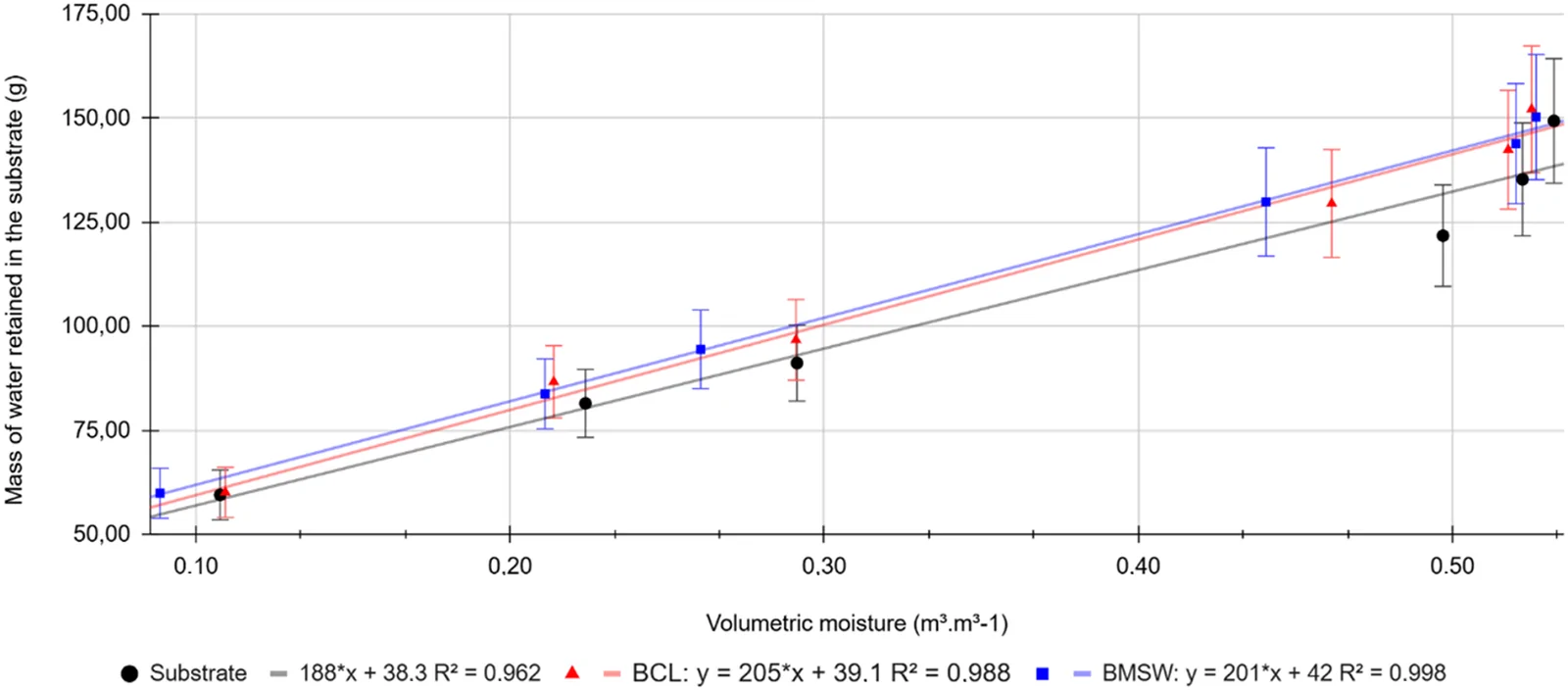
Relationship between gravimetric water mass and volumetric moisture for a commercial fertilized substrate containing municipal solid waste biochar and lemongrass biomass. Relationship between volumetric moisture (m^3^·m^–3^) and the mass of water retained in the substrate (g) for a commercial control substrate and a substrate with the incorporation of lemongrass biochar (BCL) and municipal solid waste (BMSW). The error bars represent the standard deviation of the replicates. *R* ^2^: coefficient of determination of the generated equation. *Y*-axis = mass of water retained in the substrate (g); *x*-axis = volumetric moisture of the substrate (m^3^·m^–3^).

The graph shows the relationship between the volumetric moisture content of the substrate (m^3^·m^–3^) and the mass of water retained in the substrate (g) for the different treatments evaluated (control substrate and substrates fertilized with lemongrass biochar and municipal solid waste [MSW] in different proportions).

Analysis of the slope coefficients of the regression equations indicates that the variation in the mass of water retained in the substrate as a function of volumetric moisture followed a similar pattern across treatments, with values ranging approximately from 188 to 205. This proximity suggests that the addition of biochar, at the evaluated proportions, did not result in significant changes in the substrate’s water behavior, indicating stability in the relationship between water storage and the system’s volumetric moisture.

Although the classic physical-hydric parameters, except for volumetric moisture, did not differ statistically between treatments, the integrated analysis of the fitted curves allowed us to estimate the operationally available water in the substrate, a relevant parameter for water management in container cultivation systems. In this study, this availability was operationally estimated from the relationship between water mass and volumetric moisture obtained during saturation and gravity drainage. This parameter has a direct relationship with irrigation management, as it represents a practical estimate of the water potentially available to the plants and, consequently, of the system’s water efficiency.

The approach adopted enabled a practical estimate of the available water in the substrate-plant system under controlled experimental conditions. In general, the linear regression equations showed high linearity for all treatments, with coefficients of determination ranging from 0.962 to 0.998, indicating a strong linear relationship between volumetric moisture and the mass of water retained in the substrate. This type of adjustment can help estimate operationally available water and plan water management in systems that use substrates containing biochar.

It can be observed that, in all treatments, there is a consistent linear relationship between volumetric moisture and the mass of water retained in the substrate. This behavior indicates that an increase in volumetric water content results in a proportional increase in the mass of water stored, demonstrating a predictable, homogeneous physical behavior of the substrate-water system, regardless of the source material or the applied biochar dose. Therefore, this approach may represent a simplified methodological alternative for evaluating the water behavior of substrates under experimental conditions.

From a physical-hydrological perspective, the observed linearity may indicate that, within the studied moisture range, water retention occurred due to the total volume of filled pores rather than to significant changes in pore distribution induced by biochar addition to the substrate. This suggests that the commercial substrate used already has a structure favorable to water retention, limiting the potential response to the addition of biochar in organic or fine-textured substrates.^2,35^

Scanning electron microscopy micrographs (Figures 1 and 2) show the presence of porous structures distributed on the surface of the biochar particles. This irregular, porous morphology may favor water storage in intraparticle pores, thereby contributing to capillary retention in the substrate-biochar system. However, given the biochar proportions used in this study (a maximum of 10 wt %), it is likely that the effect of these porous structures was partially diluted by the high initial porosity of the commercial substrate, which helps explain the small differences observed in the physical-hydraulic parameters.

Studies indicate that biochar’s effects on water retention are more pronounced in sandy soils or soils with low initial water retention capacity. At the same time, in substrates with high porosity and organic matter content, the gains tend to be more discrete or insignificant.^2,26,35^

Thus, the high coefficients of determination across all treatments indicate that the linear model adequately describes the relationship between volumetric moisture and the mass of water retained in the substrate over the evaluated moisture range. This behavior suggests that, under the adopted experimental conditions, the variation in water mass in the system can be estimated with good precision from volumetric moisture measurements with the TDR probe.

This pattern also indicates stability in the physical behavior of the substrate-biochar system during saturation and drainage, suggesting that the addition of biochar at the evaluated proportions did not promote significant structural changes in the substrate’s water retention dynamics. The results demonstrate that, in highly porous commercial substrates, the benefits provided by biochar tend to be more modest than those typically observed in mineral soils, reinforcing that the effect of biochar depends on the physical characteristics of the growing medium.

## 4 Conclusion

The incorporation of biochars produced from lemongrass biomass and municipal solid waste into the commercial substrate did not result in significant changes in most of the evaluated physical-hydraulic parameters. Volumetric moisture varied between 0.43 m^3^·m^–3^ and 0.53 m^3^·m^–3^, with punctual increases associated with the addition of biochars. At the same time, electrical conductivity remained at low levels (0.31–0.38 mS·cm^–1^), indicating no significant increase in substrate salinity. The container capacity after gravity drainage increased at the highest biochar doses, reaching 3.57 g_H_2_O_·g_substrate_^–1^ in the treatment with 10 wt % lemongrass biochar, suggesting a moderate contribution of these materials to water retention. The regression equations showed high linearity (R^2^ between 0.962 and 0.998), indicating stable water behavior of the substrate-biochar system, in which water retention is mainly associated with the total volume of filled pores, demonstrating the potential to operationally estimate water availability in substrates through volumetric moisture measurements using TDR sensors, which can support irrigation management strategies. The characterization of the biochars revealed alkaline materials containing mineral nutrients and concentrations of potentially toxic metals below the limits established for agricultural use, highlighting their potential use as substrate conditioners and as an alternative for the sustainable use of organic and urban waste. For future research, it is recommended to evaluate the performance of biochars in different types of commercial substrates, as well as to investigate their effects on the growth, development, and water-use efficiency of various plant species. Furthermore, studies under commercial production conditions could validate the applicability of the proposed operational water retention curve as a tool for monitoring water availability and managing irrigation in container-based cultivation systems.
